# Gene model for the ortholog of *Glys* in *Drosophila simulans*

**DOI:** 10.17912/micropub.biology.001168

**Published:** 2025-01-07

**Authors:** Madeline L. Gruys, Madison A. Sharp, Zachary Lill, Caroline Xiong, Amy T. Hark, James J. Youngblom, Chinmay P. Rele, Laura K Reed

**Affiliations:** 1 The University of Alabama, Tuscaloosa, AL USA; 2 Muhlenburg College, Allentown, PA USA; 3 California State University Stanislaus, Turlock, CA USA; 4 Muhlenburg College, Allentown, PA USA

## Abstract

Gene model for the ortholog of glycogen synthase ( *Glys* ) in the *Drosophila simulans* May 2017 (Princeton ASM75419v2/DsimGB2) Genome Assembly (GenBank Accession: GCA_000754195.3 ). This ortholog was characterized as part of a developing dataset to study the evolution of the Insulin/insulin-like growth factor signaling pathway (IIS) across the genus *Drosophila* using the Genomics Education Partnership gene annotation protocol for Course-based Undergraduate Research Experiences.

**Figure 1. Glys gene model comparison between Drosophila simulans and Drosophila melanogaster orthologs:**
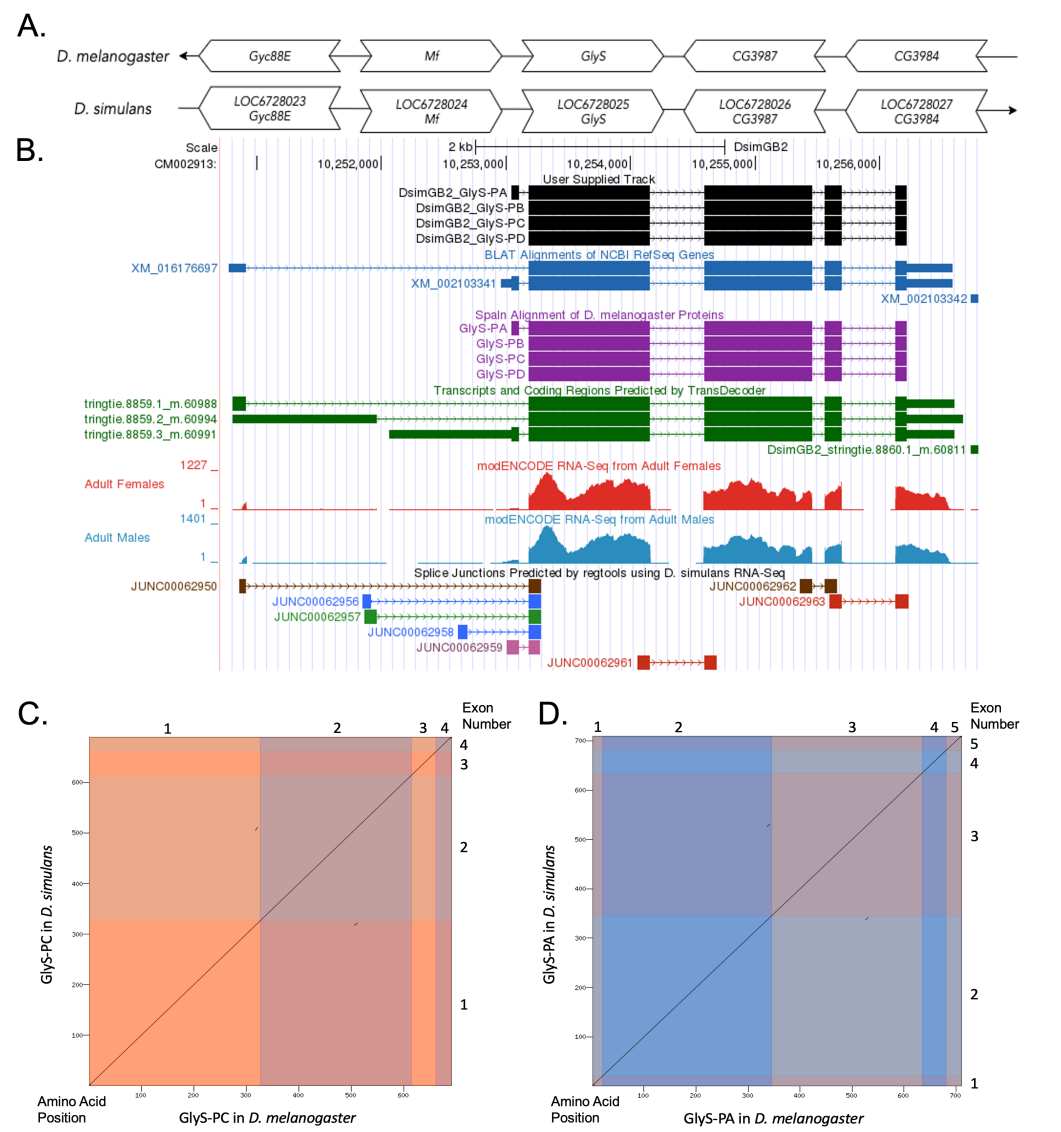
**
(A) Synteny comparison of the genomic neighborhoods for *Glys* in *Drosophila melanogaster* and *D. simulans* .** Thin underlying arrows indicate the DNA strand within which the target gene– *Glys* –is located in *D. melanogaster* (top) and *D. simulans* (bottom) genomes. The thin arrow pointing to the right indicates that *Glys* is on the positive (+) strand in *D. simulans* , and the thin arrow pointing to the left indicates that *Glys* is on the negative (-) strand in *D. melanogaster* . The wide gene arrows pointing in the same direction as *Glys* are on the same strand relative to the thin underlying arrows, while wide gene arrows pointing in the opposite direction of *Glys* are on the opposite strand relative to the thin underlying arrows.White gene arrows in *D. simulans* indicate orthology to the corresponding gene in *D. melanogaster* . Gene symbols given in the *D. simulans* gene arrows indicate the orthologous gene in *D. melanogaster* , while the locus identifiers are specific to *D. simulans* . **(B) Gene Model in GEP UCSC Track Data Hub (Raney et al., 2014).** The coding-regions of *Glys* in *D. simulans* are displayed in the User Supplied Track (black); CDSs are depicted by thick rectangles and introns by thin lines with arrows indicating the direction of transcription. Subsequent evidence tracks include BLAT Alignments of NCBI RefSeq Genes (dark blue, alignment of Ref-Seq genes for *D. simulans* ), Spaln of D. melanogaster Proteins (purple, alignment of Ref-Seq proteins from *D. melanogaster* ), Transcripts and Coding Regions Predicted by TransDecoder (dark green), RNA-Seq from Adult Females and Adult Males (red and light blue, respectively; alignment of Illumina RNA-Seq reads from *D. simulans* ), and Splice Junctions Predicted by regtools using *D. simulans* RNA-Seq (SRP006203). Splice junctions shown have a read-depth of 10-49, 50-99, 100-499, 500-999, >1000 supporting reads in blue, green, pink, brown, and red, respectively. **(C) Dot Plot of Glys-PC in *D. melanogaster* ( *x* -axis) vs. the orthologous peptide in *D. simulans* ( *y* -axis).** Amino acid number is indicated along the left and bottom; CDS number is indicated along the top and right, and CDSs are also highlighted with alternating colors. ( **D) Dot Plot of Glys-PA in *D. melanogaster* (x-axis) vs. the orthologous peptide in *D. simulans* (y-axis).** Amino acid number is indicated along the left and bottom; CDS number is indicated along the top and right, and CDSs are also highlighted with alternating colors.

## Description

**Table:** 

*This article reports a predicted gene model generated by undergraduate work using a structured gene model annotation protocol defined by the Genomics Education Partnership (GEP; thegep.org) for Course-based Undergraduate Research Experience (CURE). The following information in this box may be repeated in other articles submitted by participants using the same GEP CURE protocol for annotating Drosophila species orthologs of Drosophila melanogaster genes in the insulin signaling pathway.* "In this GEP CURE protocol students use web-based tools to manually annotate genes in non-model *Drosophila* species based on orthology to genes in the well-annotated model organism fruitfly *Drosophila melanogaster* . The GEP uses web-based tools to allow undergraduates to participate in course-based research by generating manual annotations of genes in non-model species (Rele et al., 2023) . Computational-based gene predictions in any organism are often improved by careful manual annotation and curation, allowing for more accurate analyses of gene and genome evolution (Mudge and Harrow 2016; Tello-Ruiz et al., 2019) . These models of orthologous genes across species, such as the one presented here, then provide a reliable basis for further evolutionary genomic analyses when made available to the scientific community.” (Myers et al., 2024) . “The particular gene ortholog described here was characterized as part of a developing dataset to study the evolution of the Insulin/insulin-like growth factor signaling pathway (IIS) across the genus *Drosophila* . The Insulin/insulin-like growth factor signaling pathway (IIS) is a highly conserved signaling pathway in animals and is central to mediating organismal responses to nutrients (Hietakangas and Cohen 2009; Grewal 2009) .” (Myers et al., 2024) . “ *D. simulans* is part of the *melanogaster* species group within the subgenus *Sophophora* of the genus *Drosophila* (Sturtevant 1939; Bock and Wheeler 1972) *.* It was first described by Sturtevant (1919). *D. simulans* is a sibling species to *D. melanogaster* , thus extensively studied in the context of speciation genetics and evolutionary ecology (Powell 1997) . Historically, *D. simulans* was a tropical species native to sub-Saharan Africa (Lemeunier et al., 1986) where figs served as a primary host (Lachaise and Tsacas 1983) . However, *D. simulans's* range has expanded worldwide within the last century as a human commensal using a broad range of rotting fruits as breeding sites (https://www.taxodros.uzh.ch, accessed 1 Feb 2023).” (Lawson et al., 2024) . “ *Glycogen synthase* ( *Glys* ; aka. *GS, GlyS* ) is a gene within the Insulin-signaling pathway in *Drosophila* and encodes a glycosyltransferase that catalyzes linkage of glucose monomers into glycogen. Glys activity is regulated allosterically by glucose 6-phosphate and phosphorylation/dephosphorylation allowing for control of cellular glycogen levels (Plyte et al., 1992; Roach et al., 2012) . Null *Glys* mutants exhibit growth defects and reduced larval viability in *Drosophila* (Yamada et al., 2019) .” (Backlund et al., 2024) .

We propose a gene model for the *D. simulans* ortholog of the *D. melanogaster* Glycogen synthase ( *Glys* ) gene. The genomic region of the ortholog corresponds to the uncharacterized protein LOC6728025 (RefSeq accession XP_016034640.1 ) in the ASM75419v3 Genome Assembly of *D. simulans* (GenBank Accession: GCA_000754195.3 - Hu et al., 2013). This model is based on RNA-Seq data from *D. simulans* ( SRP006203 - Graveley et al., 2011) and *Glys* in *D. melanogaster* using FlyBase release FB2022_04 ( GCA_000001215.4 ; Larkin et al., 2021). Gene and species details can be found in the description above.

***Synteny***

The reference gene, *Glys* , occurs on chromosome 3R in *D. melanogaster* and is flanked upstream by *Guanylyl cylase at 88E* ( *Gyc88E* ) and *Myofilin* ( *Mf* ) and downstream by *CG3987* and *CG3984 .* The *tblastn* search of *D. melanogaster* Glys-PC (query) against the *D. simulans* (GenBank Accession: GCA_000754195.3 ) Genome Assembly (database) placed the putative ortholog of *Glys* within scaffold CM002913 (CM002913.1) at locus LOC6728025 ( XP_016034640.1 )— with an E-value of 0.0 and a percent identity of 100.00%. Furthermore, the putative ortholog is flanked upstream by LOC6728023 ( XP_016034621.1 ) and LOC6728024 ( XP_044779336.1 ), which correspond to *Gyc88E* and *Mf* in *D. melanogaster* (E-value: 0.0 and 0.0; identity: 97.27% and 97.82%, respectively, as determined by *blastp* ; Figure 1A, A ltschul et al., 1990). The putative ortholog of *Glys* is flanked downstream by LOC6728026 ( XP_002103378.1 ) and LOC6728027 ( XP_016034642.1 ), which correspond to *CG3987* and *CG3984* in *D. melanogaster* (E-value: 0.0 and 6e-168; identity: 87.14% and 85.13%, respectively, as determined by *blastp* ). The putative ortholog assignment for *Glys* in *D. simulans* is supported by the following evidence: The genes surrounding the *Glys* ortholog are orthologous to the genes at the same locus in *D. melanogaster* and local synteny is completely conserved, supported by results generated from *blastp* , so we conclude that LOC6728025 is the correct ortholog of *Glys* in *D. simulans* ( Figure 1A ).

***Protein Model***

*Glys* in *D. simulans* has two unique protein-coding isoforms (Glys-PA and Glys-PC, identical to Glys-PB and Glys-PD; Figure 1B ). mRNA isoforms Glys-RC, Glys-RB, and Glys-RD, which differ in their UTRs, contain four CDSs ,and isoform Glys-RA contains five CDSs. Relative to the ortholog in *D. melanogaster* , the RNA CDS number is conserved. The sequence of Glys-PC in *D. simulans* has 100.00% identity (E-value: 0.0) with the protein-coding isoform Glys-PC in *D. melanogaster* , as determined by *blastp* ( Figure 1C ). The dot plot of the ortholog with Glys-PA is exhibited in Figure 1D (E-value: 0.0; identity: 100.00%, as determined by *blastp* . Coordinates of this curated gene model (Glys-PA, Glys-PB, Glys-PC, Glys-PD) are stored by NCBI at GenBank/BankIt (accession **BK064593 , BK064594 , BK064595 , BK064596** , respectively). These data are also archived in the CaltechDATA repository (see “Extended Data” section below).

## Methods

Detailed methods including algorithms, database versions, and citations for the complete annotation process can be found in Rele et al. (2023). Briefly, students use the GEP instance of the UCSC Genome Browser v.435 (https://gander.wustl.edu; Kent WJ et al., 2002; Navarro Gonzalez et al., 2021) to examine the genomic neighborhood of their reference IIS gene in the *D. melanogaster* genome assembly (Aug. 2014; BDGP Release 6 + ISO1 MT/dm6). Students then retrieve the protein sequence for the *D. melanogaster* reference gene for a given isoform and run it using *tblastn* against their target *Drosophila* species genome assembly on the NCBI BLAST server (https://blast.ncbi.nlm.nih.gov/Blast.cgi; Altschul et al., 1990) to identify potential orthologs. To validate the potential ortholog, students compare the local genomic neighborhood of their potential ortholog with the genomic neighborhood of their reference gene in *D. melanogaster* . This local synteny analysis includes at minimum the two upstream and downstream genes relative to their putative ortholog. They also explore other sets of genomic evidence using multiple alignment tracks in the Genome Browser, including BLAT alignments of RefSeq Genes, Spaln alignment of *D. melanogaster* proteins, multiple gene prediction tracks (e.g., GeMoMa, Geneid, Augustus), and modENCODE RNA-Seq from the target species. Detailed explanation of how these lines of genomic evidenced are leveraged by students in gene model development are described in Rele et al. (2023). Genomic structure information (e.g., CDSs, intron-exon number and boundaries, number of isoforms) for the *D. melanogaster* reference gene is retrieved through the Gene Record Finder (https://gander.wustl.edu/~wilson/dmelgenerecord/index.html; Rele et al *.,* 2023). Approximate splice sites within the target gene are determined using *tblastn* using the CDSs from the *D. melanogaste* r reference gene. Coordinates of CDSs are then refined by examining aligned modENCODE RNA-Seq data, and by applying paradigms of molecular biology such as identifying canonical splice site sequences and ensuring the maintenance of an open reading frame across hypothesized splice sites. Students then confirm the biological validity of their target gene model using the Gene Model Checker (https://gander.wustl.edu/~wilson/dmelgenerecord/index.html; Rele et al., 2023), which compares the structure and translated sequence from their hypothesized target gene model against the *D. melanogaster* reference gene model. At least two independent models for a gene are generated by students under mentorship of their faculty course instructors. Those models are then reconciled by a third independent researcher mentored by the project leaders to produce the final model. Note: comparison of 5' and 3' UTR sequence information is not included in this GEP CURE protocol.

## Data Availability

Description: File contains the GFF, aminio acid, and nucleic acid sequences for the gene model.. Resource Type: Model. DOI: https://doi.org/10.22002/ywzts-78198
